# Baseline Body Mass Predicts Average Depressive Symptoms over the Next Two Decades for White but Not Black Older Adults

**DOI:** 10.3390/geriatrics4010014

**Published:** 2019-01-18

**Authors:** Shervin Assari

**Affiliations:** 1 Department of Psychiatry, University of Michigan, Ann Arbor, MI 48109, USA; 2 Center for Research on Ethnicity, Culture and Health, School of Public Health, University of Michigan, Ann Arbor, MI 48109, USA; 3 Department of Psychology, University of California Los Angeles (UCLA), Los Angeles, CA 90095, USA

**Keywords:** ethnic groups, whites, blacks, obesity, depressive symptoms, depression, depressive symptoms, older adults, elderly

## Abstract

Background: Although obesity and depression have a bidirectional association, this link may vary based on race. The current study tested racial variation in bidirectional links between depressive symptoms and body mass index (BMI) over 24 years of follow-up in older adults over the age of 50 in the United States. We hypothesized weaker bidirectional links in Blacks compared to Whites. Methods: Data came from waves 1 to 12 (1990 to 2014) of the Health and Retirement Study (HRS), an ongoing state-of-the-art national cohort. The study followed a representative sample of Americans (n = 15,194; 2,200 Blacks and 12,994 Whites) over the age of 50. Dependent variables were average depressive symptoms and BMI over 24 years, based on measurements every other year, from 1990 to 2014. Independent variables included baseline depressive symptoms and BMI. Covariates included age, gender, marital status, veteran status, and activities of daily living. Structural equation models were fitted to the data for data analysis. Results: In the pooled sample, bidirectional associations were found between BMI and depressive symptoms as baseline BMI predicted average depressive symptoms over time and baseline depressive symptoms predicted average BMI over 24 years. Racial differences were found in the bidirectional association between BMI and depressive symptoms, with both directions of the associations being absent for Blacks. For Whites, baseline BMI predicted average depressive symptoms over the next 24 years. Conclusion: Reciprocal associations between BMI and depressive symptoms over a 24-year period among individuals over the age of 50 vary for Blacks and Whites. As these associations are stronger for Whites than Blacks, clinical and public health programs that simultaneously target comorbid obesity and depression may be more appropriate for Whites than Blacks.

## 1. Introduction 

Obesity and depression are two comorbid pressing public health problems in the United States [1]. Obesity reduces sense of well-being and increases risk of chronic disease and mortality [2]. Depression and depressive symptoms reduce quality of life and increase risk of morbidity and mortality [3].

Recent research suggests that social, psychosocial, and medical factors that correlate with obesity may depend on race and ethnicity [4,5,6], gender [4,7], and their intersection [8]. Less is known, however, about racial differences in correlates of obesity in older adults. Information regarding racial differences in correlates of obesity can inform public and clinical practice. Such information has implications for policies and programs that aim to reduce burden of obesity across racial minorities, who are at an increased risk for obesity [9].

Correlates of depression and depressive symptoms also depend on race, gender, and their intersection [10]. For instance, Blacks and Whites differ in how depression is associated with socioeconomic status (SES) [11,12,13], medical conditions [10], and inflammation [14,15]. Depression is associated with different levels of positive and negative cognitions and emotions in Whites and Blacks [16,17,18]. Blacks with depression maintain high levels of mastery [17], hope [19], and positive emotions [18]; a pattern that is seen in elderly [17] and youth [18]. High concordance of positive and negative emotions in Blacks [19,20] may explain why negative emotions including depression [21,22,23,24,25,26,27,28,29] increase risk of medical disease [27,28,29,30,31] in Whites, but not Blacks. 

Race also alters the association between depression and obesity [30,31]. One example is the reverse link between depression and obesity in Black men [31]. In another study, the positive link between sustained depression and obesity was limited to White women, a pattern that was absent for Black women, Black men, and White men [32]. In another study, the intersection of race, age and gender altered correlates of BMI [10]. Finally, among people with obesity, the level of obesity had a weaker effect on depression for Black women, compared to White women [33]. 

Race, ethnicity, culture, and SES alter the emotional and behavioral consequences of medical conditions [34,35,36,37], and obesity is not an exception to this rule [38,39]. Whites and Blacks may vary in how poor physical health influences their mental health [34,40,41,42,43,44]. Biological correlates of negative emotions are also dependent on culture, SES, and ethnicity [36]. To be more specific, White Americans may have stronger biological correlates of negative emotions such as depression [45] and anger [35,37]. Blacks and Whites also differ in how negative emotions alter blood pressure [46]. In line with these studies, a number of epidemiological studies have documented a better predictive role of negative emotions on deterioration of physical health for Whites than Blacks [41,42,43,44,47].

The Black–White mental health paradox is still an unexplained phenomenon in public health. According to this paradox, despite the disproportionately higher rate of social, economic, and medical adversities, stressors, and risk factors, and lower SES [48,49,50], Blacks less frequently display diagnostic criteria for depression, and report better well-being than Whites [51,52]. 

One potential explanation for the weaker effects of adversities and risk factors for Blacks is that their systematic resilience has enabled them to maintain psychological well-being. Blacks may have gained psychological resilience from their life experiences, living under adversities, and experiencing multiple physical health issues [19,53]. Habituation and particular coping mechanisms may have helped Blacks gain a systemic resilience, meaning reduced effects of any additional stress on mental well-being and depression [54].

Culture and SES also suggest that obesity may cause more depression for Whites than Blacks [55]. Minorities, such as Blacks and low SES groups, may have a higher tolerance for larger body sizes and higher BMI [56]. As a result, obesity may result in lower levels of body dissatisfaction and negative body image perception for minorities, resulting in lower risk of comorbid depression among individuals with obesity [57,58,59]. The effect of high BMI on body image perception and body dissatisfaction [59,60,61,62,63,64,65] depends on race and ethnicity [66,67,68,69,70,71,72,73]. As a result, Black women with higher BMI may maintain positive self-image [74,75,76,77], which may be a cultural phenomenon [78,79,80,81]. Blacks with higher BMI may also receive affirmations, while Whites with obesity may experience expectations to be thin from their social network [78,80] and the media [78,79]. Media also over-emphasizes the thin body ideals for Whites, particularly White women [66,78,81].

Despite the existing research on racial differences [10,33] in the bidirectional links between obesity and depression in youth and adults [82,83,84,85,86], less is known about such racial differences over a long period of time among older adults. This study compared Black and White older adults for the bidirectional associations between baseline and average BMI and depressive symptoms over a 24-year period in a national longitudinal cohort study of adults over age of 50 in the U.S. In line with previous research [7,11,31,32], we hypothesized weaker links in Blacks compared to Whites. That is, baseline BMI would show stronger associations with average depression over time for Whites, and vice versa, compared to Blacks.

## 2. Methods

### 2.1. Setting and Design

With a longitudinal cohort design, data from the Health and Retirement Study (HRS) (1990–2014) were used to study the bidirectional associations between BMI and depressive symptoms over time [11]. The HRS is a state-of-the-art longitudinal panel study of a nationally representative sample of American adults 50 years or older. The study began in 1990 and is still ongoing. More information regarding the HRS methodology and sampling is published elsewhere [87,88]. 

### 2.2. Ethics

The HRS protocol was approval by the University of Michigan Institutional Review Board (IRB). All study participants signed a written consent form. They were also compensated for their participation in the study. 

### 2.3. Participants and Sampling

**Figure 1:**
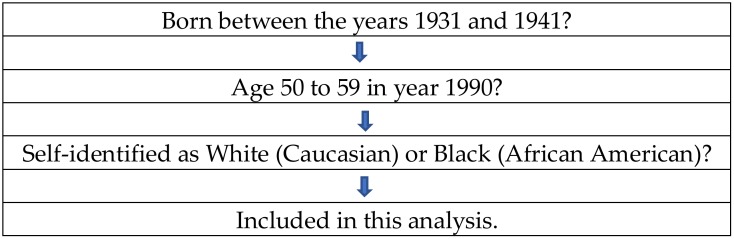
The flowchart of participants’ selection.

HRS participants who were entered into this analysis were born between the years 1931 and 1941. This study consisted of individuals in the HRS waves 1 (year 1990) to 12 (year 2014). The current study was limited to individuals who self-identified as White (Caucasian) or Black (African American). At baseline (1990), there was a total of 15,194 participants, composed of 2,200 Blacks and 12,994 Whites. These individuals entered our analysis, regardless of attrition in the future waves (because we could calculate an average even when there was only one observation). Figure 1 shows a flowchart of participants’ selection. 

### 2.4. Process

Data were collected in the face-to-face or and telephone interviews. Data were collected using proxy interviews for the participants who were unable to respond for themselves. HRS collects extensive demographic, economic, social, behavioral, psychological, and health data every two years.

### 2.5. Measures

*Race/Ethnicity*: Survey respondents self-identified their race as Black/African American or White/European. Self-identification is more valid for racial groups (Black versus White) than ethnicity (Hispanics versus non-Hispanics). This measurement approach is accepted and commonly used in social sciences and national epidemiological surveys, even if it can introduce some levels of bias (misclassification). Self-identification of race is well accepted in social sciences.

The study measured race/ethnicity, age (years), gender (male 0, female 1), marital status, veteran status, activities of daily living (ADL), BMI, and depressive symptoms.

Activities of Daily Living (ADL): HRS measured ADL using the following items: “Because of a health or memory problem do you have any difficulty with [ADL]”?, where [ADL] referred to six distinct activities, namely: (1) dressing, (2) walking across a room, (3) bathing or showering,(4) eating, (5) getting in and out of bed, and (6) using the toilet.

Body Mass Index (*BMI*): Body mass index was measured using participants’ self-reported height and weight. Height was measured in feet and inches. Weight was measured in pounds. Height and weight were converted to meters and kilograms to calculate BMI. BMI was estimated by dividing weight (kilograms) by height squared (meters squared). BMI based on self-reported height and weight has been validated in previous research [89]; however, validity of self-reported BMI may be lower for older than younger adults [90]. 

Depressive Symptoms: The eight-item Center for Epidemiologic Studies–Depression scale (CES-D) was applied to measure severity of depressive symptoms, starting in 1994. The CES-D is a self-reported measure that asks about the frequency of depressive symptomatology over the past week [91]. A CES-D score was calculated for individuals by taking the average of the items. A higher score indicated more frequent depressive symptomatology [91]. 

### 2.6. Statistical Analysis

Univariate and bivariate analysis were done in SPSS 21.0 (IBM Inc., Armonk, NY, USA). Pearson’s correlation and independent samples Student *t*-tests were used for bivariate associations. We used AMOS 18.0 (IBM Corp. Armonk, NY, USA) to conduct multivariable analysis [92,93]. 

Structural equation modeling (SEM) was used for multivariable data analysis [94]. In the first step, a model was run in the pooled sample. Then we ran multi-group SEM analysis, in which the groups were defined based on race. 

We fitted models with and without constrained paths across the groups. As the fit did not improve in the presence of constraints, we reported the model without constraints. We also tested models with and without error variances for the outcomes. The fit dramatically enhanced with the errors correlated, so we kept the correlation between the errors of the outcomes in the final model. 

Independent variables included baseline depressive symptoms and baseline BMI. The two dependent variables included average depressive symptoms and average BMI between 1990 and 2014, based on measurements every two years. Covariates included age, gender, marital status, veteran status, and activities of daily living. 

Paths (effects) were conceptualized from all the covariates to the dependent variables and also from independent variables to dependent variables. The path from veteran status to average BMI was omitted as the model could not be saturated. Errors of dependent variables were allowed to covary. 

Fit statistics included the chi square (CMIN), the root mean squared error of approximation (RMSEA), the comparative fit index (CFI), and the X2 to degrees of freedom ratio (CMIN/DF) [95,96,97]. Standardized regression coefficients and their standard errors (SE) and associated *p* values were reported. *P*-values less than 0.05 were considered significant.

Attrition: Attrition had a minimal effect in this study for several reasons. First, AMOS applies full information maximum likelihood (FIML) to handle missingness. Thus, the analysis is not limited to individuals with complete data, and any available data are used in the modeling (SEM). In addition, attrition was not a reason for excluding individuals from the modeling because one observation is enough for calculation of average. This approach was taken because in long-term follow-up studies, cumulative attrition is high, and is a major source of bias. In our study, however, any person who had data at baseline entered the analysis.

## 3. Results

### 3.1. Univariate Analysis

Table 1 describes the pooled sample as well as Blacks and Whites. As shown in the table, the White sample had a higher percentage of men than did the Black sample. While Whites and Blacks did not differ in age, Whites were married more often, and had better health status regardless of the domains (BMI and depressive symptoms) at baseline and over time. 

### 3.2. Bivariate Analysis

Table 2 shows the correlation matrix of the study variables in the pooled sample, as well as Whites and Blacks. In the pooled sample, race was positively correlated with high BMI and depressive symptoms at baseline, as well as average BMI and depressive symptoms during the follow-up. In the pooled sample, baseline BMI and depressive symptoms were positively correlated with average BMI and average depressive symptoms during the follow up duration. These correlations were significant in Whites, but not in Blacks. 

### 3.3. Multivariable Analysis

**Figure 2:**
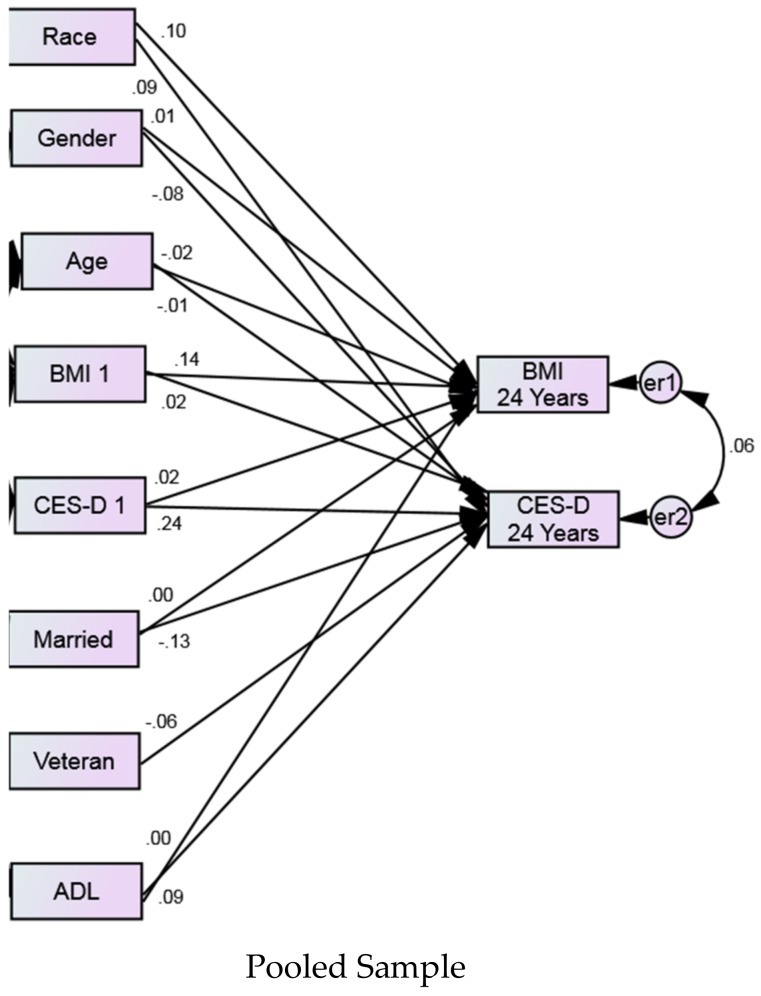
Structural equation model (SEM) in the pooled sample. Dependent variables: average depressive symptoms and BMI over 24 years. Independent variables: baseline depressive symptoms and BMI. Covariates: age, race, gender, marital status, veteran status, and ADL. Numbers reflect standardized adjusted path coefficients. Probability level = 0.274, Chi-square (CMIN) = 1.198, DF = 1, CMIN/DF = 1.198, CFI = 1.000, RMSEA = 0.002 (0.000–0.014).

Our SEM in the pooled sample showed an excellent fit (probability level = 0.274, chi-square (CMIN) = 1.198, DF = 1.000, CMIN/DF = 1.198, CFI 1.000, RMSEA 0.002 (0.000–0.014)). In the pooled sample, bidirectional associations were found between baseline and average BMI and depressive symptoms over the follow up period, as baseline BMI predicted average depressive symptoms over time and baseline depressive symptoms predicted average BMI over 24 years (Table 3 and Figure 2).

**Figure 3:**
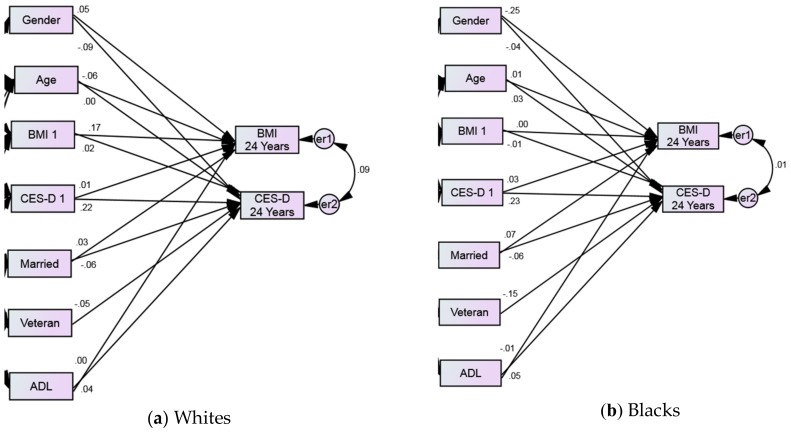
Structural equation model (SEM) based on race. Dependent variables: Average depressive symptoms and BMI over 24 years. Independent variables: baseline depressive symptoms and BMI. Covariates: age, gender, marital status, veteran status, and ADL. Numbers reflect standardized adjusted path coefficients. Probability level = 0.285, chi-square (CMIN) = 2.507, DF = 2, CMIN/DF = 1.254, CFI = 1.000, RMSEA = 0.004 (0.000–0.017).

In Whites, baseline BMI predicted average depressive symptoms over the follow up period, while baseline depressive symptoms did not predict average BMI over 24 years (Table 3 and Figure 3a). 

Baseline BMI did not predict average depressive symptoms over the follow up period and baseline depressive symptoms did not predict average BMI over 24 years for Blacks (Table 3 and Figure 3b). 

Table 3 also showed some age and gender differences between Black and White groups (both in terms of *p*-values and β’s). These include a stronger effect of age on average BMI for Whites than Blacks, and a stronger effect of gender on average BMI for Blacks than Whites. 

## 4. Discussion 

The current study compares Black and White older adults for the bidirectional correlations between baseline and average of depressive symptoms and BMI over 24 years. Although in the pooled sample, bidirectional associations were found (i.e., baseline BMI predicted average depressive symptoms over time and baseline depressive symptoms predicted average BMI during the follow up), these correlations could be detected for White but not Black older adults. Thus, the bidirectional links between depressive symptoms and BMI are not universal and depend on race. 

### 4.1. Previous Research

The finding of this study supports the results of previous studies showing differential associations between obesity and depression by race, gender, and their intersection [7,38,39,98,99,100,101,102]. Both cross-sectional [7,38,39,98,99,100,101,102] and longitudinal [32,99] studies have shown that the correlation between BMI and depressive symptoms varies by race and gender [55,98,99,100,101]. Overall, high BMI individuals are more likely to feel depressed if they are White than Black [33]. In a recent study, sustained obesity and depression tended to be comorbid in White women, but not White men, Black men, or Black women [55]. A nine-year follow-up study failed to show a bidirectional association between obesity and depression in Blacks [99]. Among individuals with obesity, a higher grade of obesity had a stronger effect on the depression of White women than Black women [33]. All these findings are in concert with the current finding and suggest that the link between depression and obesity are weaker for Blacks than Whites, regardless of setting, design, and age group. 

### 4.2. “Jolly Fat” Hypothesis 

The “jolly fat” hypothesis suggests that high BMI and obesity are not linked to depression in all social groups [102,103,104]. This hypothesis is particularly relevant to older adults [103,104,105,106,107]. However, some research has also shown this hypothesis for adolescents [105] and adults [108]. The “jolly fat” hypothesis is mostly relevant to older men [109]. In a population-based study of 2,245 individuals above age of 50 in Rancho Bernardo, California, depression was inversely associated with obesity in men, supporting the “jolly fat” hypothesis [109]. While most research on the “jolly fat” hypothesis is on East Asian men [103,104,105,106,107], the current study proposes the same phenomenon for Blacks.

### 4.3. Culture and Social Norms

Culture and ethnicity shape cognitive and emotional styles of social groups [110]. These cognitions and emotions are essential for evaluation of self and perception of obesity [8]. These cognitions have implications for the engagement of weight management behaviors in obese individuals [8,111]. Compared to their White counterparts, Blacks with obesity are more likely to feel healthy and are less likely to have concerns about medical risks [8]. As a result, Blacks with obesity show lower perceived obesity and lower intention to control weight, compared to Whites [8,112]. Such racial differences may be due to a higher risk of misperception of own body size as healthy weight in Blacks than Whites [113]. While a very minimal proportion of normal weight Whites wish to be heavier, a larger proportion of normal weight Blacks desire to be heavier [114]. 

Racial differences in culture and norms may result in lower psychological costs of obesity in Blacks, compared to Whites [37,55]. Having a small body size may be a more salient desire for Whites than Blacks [115]. As a result, Blacks, particularly Black women, maintain positive body image despite their obesity [116]. Blacks also show less internalized stigma toward obesity [116]. A considerable proportion of Blacks with obesity report that their significant others are satisfied with their size [117]. 

Black–White differences in body image ideals and perception [60,61,62,63,64,65], as well as social norms and social support [118], may explain why Blacks maintain better mental health despite higher BMI. Obesity in Blacks, particularly Black women, results in weaker negative social pressures for an ideal body image, compared to other social groups [119]. This reflects Blacks’ high acceptance of large body sizes [120]. According to James Jackson, Blacks may engage in unhealthy behaviors, such as overeating, to cope with social and economic adversities and stressors in their daily lives [120,121]. However, this pattern may defer between Black men and women. While Black men may have a higher tendency towards substance use, Black women may respond by turning to comfort food [120]. 

### 4.4. Cultural Moderation Hypothesis

Kitayama et al. have discussed the cultural moderation hypothesis. According to this hypothesis, culture determines the correlates of affect, emotion, and health [37]. Risk factors seem to be more influential on mental health when the thought system is analytical (e.g., Blacks) rather than holistic (e.g., Whites) [110]. Blacks with depression maintain higher level of hope [19], mastery [17], and positive emotions [20] than Whites with depression. According to the undoing hypothesis [20], these positive emotions and cognitions are protective and help to maintain the physical health of individuals, even in the presence of high negative emotions [20].

### 4.5. Blacks’ Resilience

Blacks demonstrate systemic resilience despite all the stressors [51,52]. Blacks report higher levels of optimism and hope than Whites under adversities [19,20,121,122]. This pattern is in contrast to the cumulative or multiple adversity hypotheses, that conceptualize Blacks and other minority groups as vulnerable groups [123]. These models suggest stronger effects of medical risk factors, such as obesity, on well-being in Blacks compared to Whites [124]. Due to historic oppression, racism, and discrimination [125,126,127], and the multiple disadvantages of Blacks, these models should predict an increase in the vulnerability of Blacks to each risk factor [128,129,130]. However, similar to the findings reported here, the effects of risk factors are commonly weaker in Blacks than Whites [131,132].

The systemic resilience that Blacks show is not specific to the effect of obesity on depression and extends to a wide range of domains and health outcomes [53]. Such systemic resilience among Blacks may reflect the context and culture of Blacks’ lives. According to the Law of Small Effects, health disparities are effects of cumulative effects of small differences due to a large number of risk factors that accumulate over the lifespan [133,134]. As a result, the contribution of each risk factor is small, as such risk is happening in the context of a variety of other risk factors [133,134]. As a result, we should not overestimate the impact of addressing each risk factor on the health of Blacks. Instead, multi-level and multi-dimensional interventions are needed [53]. 

### 4.6. Differential Effects

Race is a proxy of SES, environmental exposures, treatment by the society, and access to the opportunity structure. All these social factors may alter vulnerability of populations to certain risk factors. In a conceptual paper, Assari reviewed more than 40 original articles in which weaker effects of risk factors on health were found in Blacks, compared to Whites [131]. This pattern has been called “differential effects”, “Blacks’ diminished return”, and the “rule of smaller effects” in previous literature [132]. Based on these theories, social risk factors have stronger effects on Whites, regardless of their type [53].

### 4.7. Black–White Mental Health Paradox 

The findings of this study align with the Black–White mental health paradox, defined as Blacks’ better mental health despite higher rates of social and medical risk factors [40]. Blacks use social support [118,135,136] and religion [135,137] to maintain a high level of mental well-being. They also maintain hope and positive emotions as well as a sense of mastery even in the presence of negative emotions and depression [19,20,121,122]. Keyes has discussed the “Black advantage” in mental health, possibly due to flourishing in response to adversity [138]. Black–White differences in resilience may be due to culture or previous experience with adversity [40,51,52,139].

### 4.8. Inflammation

Obesity and other cardiometabolic conditions are pro-inflammatory states [140,141,142,143,144,145,146]. Interestingly, in line with the previous research showing a link between depression and obesity in Blacks but not Whites [10], depression is shown to be associated with inflammation in Whites but not Blacks [45]. That is, inflammation may explain racial differences in the depression–obesity risk [33]. This evidence may also explain why negative affect predicts future depression [3,147] and future chronic disease [44,47,148] in Whites but not Blacks. 

### 4.9. Implications

These results may have clinical and public health implications. Social groups have differences in patterns of comorbidity of health problems [149,150,151]. At least for Whites, this study highlights the need for a multidisciplinary and team approach to reduce the burden of obesity and depression. In this regard, there is a need to consider the context, culture, and environment of the target population when examining these factors. Programs and interventions that target prevention of obesity may have weaker effects on comorbid depression for Blacks compared to Whites. Group-specific associations between BMI and mental problems advocate for tailoring interventions across social groups. Universal interventions may have differential effects for diverse populations as Whites and Blacks with obesity have different levels of mental health need. To maximize the benefits, interventions may consider race, gender, class, and place of the target population. Weight loss interventions may differently impact White and Black men and women [152]. Some social groups who may be at risk of sub-optimal weight loss may also need additional support as part of their weight loss programs. 

### 4.10. Limitations

The current study is not without limitations. Attrition is a major source of bias in long-term cohort studies. To minimize this type of bias, we used all available participants, regardless of their attrition over time. This was because we could calculate average even in the presence of one observation. Although our study had longitudinal design, and we investigated the bidirectional associations between depression and BMI, the results only suggest association not causation. Differential rate of loss to follow-up based on race may result in racial differences in missing data and associated bias. Another limitation is the calculation of BMI based on self-reported height/weight. Although this BMI measure is valid, self-reported BMI is subject to under-estimation, particularly for women. Older adults are specifically prone to recall bias, which causes over- or under-reporting of depressive symptoms and BMI. Self-reported measures of BMI in older adults have been criticized and may cause additional bias. Other than memory problems, bone issues may confound height and validity of self-reported BMI. In addition, race was self-identified. Although this is common practice in survey research, observations could also be used in this regard. In addition, the unbalanced sample size of Whites and Blacks may have resulted in differential statistical power by race. Despite these limitations, the current study significantly contributes to the literature as it is one of the first long-term studies on the elderly. Having access to 24 years of follow-up data, in a nationally representative sample, with a large sample size are some of the key strengths of the current study. 

### 4.11. Future Research

Further research should investigate the underlying biological as well as behavioral mechanisms for racial differences in the link between BMI and depression. More research should determine the most effective multi-component interventions for each racial group. Future studies should evaluate other psychosocial factors that need to be targeted in treatment for Blacks with obesity. Research is also needed on the efficacy of tailored interventions for reduction of obesity and depression among the elderly. We took a simplistic analytical approach that did not allow modeling of timing of the changes of BMI and depression. This was because we had only hypothesized Black - White differences in the bidirectional longitudinal association between the baseline and average of CESD and BMI over time, using structural equation modeling. Future research may use other techniques such as latent growth curve, latent class analysis, lagged effects, or generalized estimating equations. Some of these techniques are helpful if researchers are interested in discovering time-period effects. Researchers may also want to stratify the analyses by year of BMI or CESD measurement to test if the associations between BMI and CESD are time-dependent, and whether such periods are differently relevant to White and Black participants. 

Another limitation was that we assumed that all White and Black groups are composed of a homogeneous sample. There are, however, multi-racial people in the US. Future research may explore such complexities by including mixed race, and considering heterogeneities by ethnicity. We only included age, gender, marital status, veteran status and ADL as covariates. Future research may also include wealth, income, education, place of residence, social capital and their possible interactions with race, BMI and depression. Finally, we pooled all older adults to one age group. The oldest adults, however, may differ in the depression–obesity link, from younger older adults. Future research may test these variations for cohorts below and over 80 years. Future research should also adjust for co-morbid chronic illnesses such as arthritis and diabetes which can impact BMI and depression. Finally, in older adults, not only high BMI but also underweight can be linked to chronic disease, disabilities, and depression. Future research may test all of these complexities.

## 5. Conclusions

In conclusion, the bidirectional associations between baseline and average of BMI and depressive symptoms over time in older adults depend on race, with weaker links being found in Blacks than Whites. Future research should test if combined programs that simultaneously address depression and obesity are more effective for Whites than Blacks. Research should also test if separate programs are more appropriate for Blacks. Both populations need additional investment in screening, diagnosis, and treatment of depression and obesity.

## Figures and Tables

**Table 1:** Descriptive statistics in the pooled sample and based on race and gender.

		All			Whites			Blacks	
Gender *	n	%		n	%		n	%	
Male	6431	42.33		5614	43.20		817	37.14	
Female	8763	57.67		7380	56.80		1383	62.86	
Veterans *									
No	4348	68.33		3775	67.09		573	77.85	
Yes	2015	31.67		1852	32.91		163	22.15	
Married *									
No	1555	20.05		1086	16.50		469	40.05	
Yes	6199	79.95		5497	83.50		702	59.95	
									
	n	Mean	SD	n	Mean	SD	n	Mean	SD
									
Age (Years)	7754	55.01	4.94	6583	55.01	4.97	1171	55.00	4.75
ADL (Count) *	9728	0.09	0.43	8338	0.08	0.40	1390	0.16	0.58
CES-D (Count)1 *	9271	1.19	1.85	7952	1.11	1.79	1319	1.69	2.11
BMI (kg/m^2^) 1 *	7754	27.14	5.04	6583	26.81	4.78	1171	29.01	6.00
Average CES-D (Count) During 24 Years *	11,684	1.25	1.39	10,381	1.20	1.35	1303	1.67	1.57
Average BMI (kg/m^2^) During 24 Years*	12,226	27.41	4.99	10,825	27.22	4.88	1401	28.85	5.61

BMI—Body mass index; ADL—activities of daily living; CES-D—Center for Epidemiologic Studies Depression scale); * *p* < 0.05.

**Table 2:** Correlation matrix of the study variables in the pooled sample and based on race.

Characteristics	1	2	3	4	5	6	7	8	9	10
All										
(1) Race	1	0.043 **	−0.001	−0.074 **	−0.211 **	0.063 **	0.109 **	0.156 **	0.107 **	0.104 **
(2) Gender		1	−0.250 **	0.616 **	−0.136 **	0.049 **	0.132 **	−0.026 *	−0.094 **	0.012
(3) Age			1	−0.119 **	−0.025 *	0.003	−0.047 **	−0.005	0.019	−0.055 **
(4) Veterans				1	0.026 *	−0.005	0.014	−0.064 **	−0.118 **	−0.003
(5) Marital Status					1	−0.075 **	−0.156 **	−0.035 **	−0.063 **	0.002
(6) ADL (count)						1	0.296 **	0.128 **	0.095 **	0.012
(7) CES-D 1 (count)							1	0.100 **	0.216 **	0.029 *
(8) BMI 1 (kg/m^2^)								1	0.062 **	0.131 **
(9) Average CES-D During 24 Years									1	0.093 **
(10) Average BMI During 24 Years (kg/m^2^)										1
**Whites**										
(2) Gender		1	−0.256 **	0.639 **	−0.114 **	0.033 **	0.125 **	−0.078 **	−0.096 **	0.052 **
(3) Age			1	−0.130 **	−0.022	0.003	−0.051 **	0.010	0.017	−0.077 **
(4) Veterans				1	0.012	−0.006	0.027	−0.069 **	−0.105 **	0.026*
(5) Marital Status					1	−0.052 **	−0.140 **	−0.001	−0.047 **	0.000
(6) ADL (count)						1	0.274 **	0.109 **	0.087 **	0.015
(7) CES-D 1 (count)							1	0.086 **	0.204 **	0.026 *
(8) BMI 1 (kg/m^2^)								1	0.059 **	0.151 **
(9) Average CES-D During 24 Years									1	0.090 **
(10) Average BMI During 24 Years (kg/m^2^)										1
**Blacks**										
(2) Gender		1	−0.216 **	0.451 **	−0.192 **	0.106 **	0.140 **	0.163 **	−0.082 **	−0.260 **
(3) Age			1	−0.051	−0.048	−0.001	−0.033	−0.074 *	0.041	0.087 *
(4) Veterans				1	0.081 *	0.023	−0.030	0.040	−0.163 **	−0.154 **
(5) Marital Status					1	−0.100 **	−0.124 **	-0.003	−0.079 *	0.057
(6) ADL (count)						1	0.357 **	0.159 **	0.110 **	−0.025
(7) CES-D 1 (count)							1	0.073 *	0.233 **	−0.013
(8) BMI 1 (kg/m^2^)								1	−0.023	−0.039
(9) Average CES-D During 24 Years									1	0.034
(10) Average BMI During 24 Years (kg/m^2^)										1

BMI—Body mass index (BMI); ADL—Activities of daily living); CES-D—Center for Epidemiologic Studies Depression scale); ** *p* < 0.001, * *p* < 0.05.

**Table 3:** Path coefficients (SEM) in the pooled sample and based on race.

				Pooled Sample			Whites			Blacks	
Cross Lagged Paths			B	SE	*p*	B	SE	*p*	B	SE	*p*
BMI 1	→	CES-D 24 Years	0.02	0.00	0.017	0.03	0.00	0.050	−0.01	0.01	0.809
CES-D 1	→	BMI 24 Years	0.02	0.03	0.055	0.01	0.04	0.459	0.03	0.10	0.391
Autoregressive Paths											
BMI 1	→	BMI 24 Years	0.14	0.01	<0.001	0.17	0.01	<0.001	0.00	0.03	0.906
CES-D 1	→	CES-D 24 Years	0.24	0.01	<0.001	0.22	0.01	<0.001	0.23	0.03	<0.001
Covariates on BMI											
Race (Blacks)	→	BMI 24 Years	0.10	0.14	<0.001	-	-	-	-	-	-
Gender (Women)	→	BMI 24 Years	0.01	0.10	0.236	0.05	0.10	<0.001	−0.25	0.33	<0.001
Age	→	BMI 24 Years	−0.02	0.01	0.132	−0.06	0.01	<0.001	0.01	0.04	0.686
Marital Status (Married)	→	BMI 24 Years	0.00	0.16	0.741	0.03	0.17	0.034	0.08	0.40	0.033
ADL 1	→	BMI 24 Years	0.00	0.07	1.000	0.04	0.04	0.002	0.05	0.10	0.158
Covariates on CES-D											
Race (Blacks)	→	CES-D 24 Years	0.09	0.04	<0.001	-	-	-	-	-	-
Gender (Women)	→	CES-D 24 Years	−0.08	0.04	<0.001	−0.09	0.04	<0.001	−0.04	0.12	0.317
Age	→	CES-D 24 Years	−0.01	0.00	0.372	0.00	0.00	0.945	0.03	0.01	0.487
Marital Status (Married)	→	CES-D 24 Years	−0.13	0.05	<0.001	−0.06	0.05	<0.001	−0.06	0.12	0.089
Veteran	→	CES-D 24 Years	−0.06	0.05	<0.001	−0.05	0.05	0.002	−0.15	0.16	<0.001
ADL 1	→	CES-D 24 Years	0.09	0.02	<0.001	0.00	0.15	0.831	−0.01	0.34	0.743

BMI—Body mass index; ADL—Activities of daily living; CES-D—Center for Epidemiologic Studies Depression scale.
